# An integrated school health programme

**Published:** 2022-09-20

Lack of adherence to treatment and to the use of spectacles is a common problem in many low- and middle-income countries, which is often the result of lack of knowledge of eye health as well as negative perceptions, attitudes, and misconceptions about wearing spectacles.^1^

Using a targeted social and behaviour change approach, the school health integrated programme in Liberia, implemented between 2019-2021, aimed to provide vision screening to 76,000 children of school age. However, nearly 123,000 children were screened (far more than expected) and 590 received corrective glasses. This outcome was achieved by clearly identifying our target audience and the behaviour we needed to address, and by using local content and approaches. The lessons from Sightsavers’ school health integrated programme in Liberia, and a similar programme in Pakistan, showed that understanding why girls and boys wear (or do not wear) spectacles will help us to design effective behaviour change interventions that address barriers to their use. Some of the common barriers include:

- lack of awareness
- negative perceptions
- traditional beliefs about the use of spectacles, especially by girls
- distance
- cost.

Developing appropriate information and communication materials to target the barriers will go a long way in creating awareness about eye health services and products.

Gaining community support by engaging with parents (through parent–teacher associations) and with traditional and religious leaders may help to address misconceptions and traditional beliefs.

Different operational strategies should be employed to address the problem of distance to eye care services. For example, to reduce the distance that children may have to travel for refraction and spectacles, a central location can be designated for eye health services within a cluster of schools.

Cost recovery mechanisms can be put in place to ensure that services and spectacles are affordable for everyone.

Considering the large population of out-of-school children in low- and/or middle-income countries, these programmes also sought to identify the best approaches for encouraging both enrolled and non-enrolled school-aged children to participate in vision screening and deworming activities. Approaches included the use of peer groups (mostly made up of enrolled children who had been screened and received their deworming tablets) to encourage non-enrolled children to access the service. Engagement with community-based organisations, community volunteers, and town criers can also helped to spread information.

**Figure:**
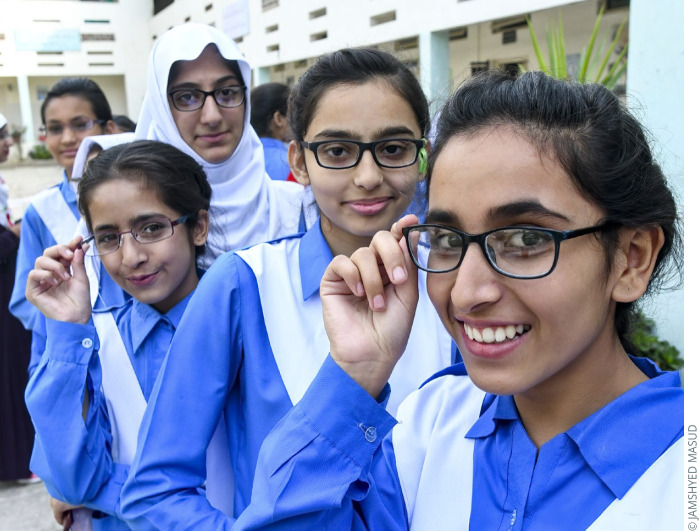
Social behaviour change interventions focused on ensuring adherence to the use of spectacles at at a school in Pakistan. pakistan

## References

[B1] Avis W. (2016). Methods and approaches to understanding behaviour change (GSDRC Helpdesk Research Report 1,389). Birmingham, UK: GSDRC, University of Birmingham. Available from: **https://bit.ly/3Qx0oDf**

